# Psychiatric Commitment: Sixty Years Under the Scrutiny of the European Court of Human Rights

**DOI:** 10.3389/fpsyt.2021.656791

**Published:** 2021-05-04

**Authors:** Gérard Niveau, Camille Jantzi, Tony Godet

**Affiliations:** University Center of Legal Medicine, Geneva University Hospitals, Geneva, Switzerland

**Keywords:** psychiatric commitment, involuntary hospitalization, human rights, ECtHR, forensic psychiatry

## Abstract

**Background and Aims:** In the field of mental health, the fundamental right to liberty is a point of tension between the practice of psychiatric commitment on the one hand and the universal concept of human rights on the other. The European Court of Human Rights (ECtHR) is a very specific means of safeguarding human rights because it allows an individual to not only assert their rights but also compel a state to bring its legislation into conformity with the principles of the European Convention on Human Rights. The aim of this study was to gather the case-law of the ECtHR on psychiatric commitment over the last 60 years and to determine how this case-law has affected national legislation and therefore psychiatric practice.

**Methods:** Jurisprudence data were collected from the HUDOC ECtHR database, and the direct effects of the ECtHR judgements on the legislations of the countries concerned were collected from the HUDOC EXEC database of the Council of Europe. The case-law of the Court included 118 judgements and 56 decisions and concerned 31 of the 45 countries that have ratified the Convention.

**Results:** This study therefore showed a direct effect of the Court's case-law on the legislation on psychiatric commitment in the various countries that have ratified the Convention. It was also possible to detect an indirect effect of this case-law through the directives of international institutions such as the directives of the Committee of Ministers of the Council of Europe concerning respect for people with mental disorders.

**Conclusions:** The ECtHR case-law therefore has a major influence on the psychiatric practice in all Council of Europe countries.

## Introduction

Liberty is a fundamental human right recognized across all democratic states. This principle is, however, subject to many exceptions that are precisely defined by the national laws of each country. Psychiatric commitment is one of these exceptions. Psychiatry is a particular medical specialty in that it uses deprivation of liberty as a therapeutic means. Therefore, according to Gostin, there is “a fundamental relationship between mental health and human rights” (1). History shows that psychiatry has often been used as a means of social repression and that cases of psychiatric abuse are numerous (2). The principle that people with mental disorders have the same rights as other citizens is relatively recent (3). All democratic states have gradually organized a set of laws that determine the conditions for the implementation of psychiatric commitment and the remedies available to patients to assert their rights and oppose the deprivation of liberty. However, the regulation of psychiatric commitment is always complex because it must allow both respect for the rights of patients and respect for public safety, including the safety of the patient himself (4). Infringing a fundamental right as a means of treatment represents a risky situation for the rights of patients (5).

When a citizen believes that he or she has had his rights violated, specifically the right to liberty during psychiatric commitment, he or she must have the ability to resort to an authority of justice to enforce his rights. In the conflict between the citizen and the State, even in democratic countries, the risk of inequality is significant. However, in countries that have ratified the European Convention of Human Rights, the European Court of Human Rights (ECtHR or the Court) gives citizens an exceptional means of defending their rights (6). The ECtHR is a very particular supranational legal system because it gives an individual the ability to not only assert his rights but also have a state condemned and, in certain cases, be at the origin of a judgement that will force the state to modify its legislation to conform to the principles of respect for fundamental rights. By filing an application with the ECtHR, a patient who has been deprived of his liberty due to psychiatric commitment can therefore not only assert his rights but also influence the national jurisdiction of the country where he or she was involuntarily hospitalized (7).

The organization of the ECtHR is relatively complex, and it has evolved over the years, both in substance and in form. Currently, 45 countries have ratified the Convention and are therefore affected by the ECtHR. An application can be made to the Court only when all domestic remedies have been exhausted. The processing of applications before the ECtHR takes place in several stages. Requests are first systematically examined for admissibility by a filtering section and a single judge. Potentially admissible applications are then examined by a one of the five Chambers of seven judges. The Chamber makes a determination on the merits as to whether the Convention has been violated. If one of the parties petitions successfully for the case to be re-heard at a higher level, the Grand Chamber becomes involved. In simplified terms, the case law of the Court therefore consists of decisions concerning the admissibility of applications and judgements concerning the violation of the articles of the Convention. A friendly settlement can occur at any time during the procedure and lead to the classification of the case (7).

If the Grand Chamber pronounces a judgement, it is transmitted to the Committee of ministers who must have it enforced. The state that is the subject of the judgement must satisfy individual obligations toward the applicant and general obligations so as to avoid a further similar violation of fundamental rights. Among these general obligations may be those that modify the legislative system, the dysfunctions of which led to the violation(s) (8).

The work of the ECtHR began in 1960; and in 2019, the organization celebrated its 60th anniversary. The aims of this study are to describe the activity of the ECtHR in the field of the legal regulation of psychiatric commitment, to expose the main cases with exemplary value and to determine which legal cases have led to changes in national legislations.

## Materials and Methods

### Case-Law of the ECtHR

All the data concerning the case-law were extracted from the HUDOC database. HUDOC is the official case-law database of the ECtHR (9). This database is open access and allows direct access to all the Court's case-law since 1960.

A search in HUDOC was carried out for the period from January 1, 1960, to December 31, 2019. The two keywords “(Art. 5-1-e) Alcoholics” and “(Art. 5-1-e) Persons of unsound mind” were used with the Boolean operator OR. The language filter was used to remove duplicates by using the “English OR French” selection.

The case-law data have been classified in accordance with the Court's method, which distinguishes four degrees of importance of decisions and judgements: the key cases, the level 1 cases, the level 2 cases and the level 3 cases. The key cases refer to situations concerning large groups of identical cases that are derived from the same underlying problem. The judgement of the Court permits to identify the dysfunction under national law that is the root of a violation and to give indications to the government to create a domestic remedy capable of dealing with similar cases. Level 1 cases, which are of high importance, refer to situations that make a significant contribution to the development, clarification or modification of the Court's case-law, either generally or in relation to a particular state. Level 2 cases, which are of medium importance, refer to situations that do not make a significant contribution to the case-law but go beyond merely applying existing case-law. Level 3 cases, which are of low importance, refer to situations of little legal interest.

### Effects of ECtHR Judgements and Decisions on Domestic Laws

To underscore the influence of the Court on national jurisdictions concerning psychiatric commitment, the measures taken by the governments of the different countries concerned by the key cases and the level 1 cases were sought. This research was carried out using the HUDOC EXEC database of the Department for the Execution of Judgements of the ECtHR of the Council of Europe (10). A specific search was carried out for each judgement or decision by referring to the case code number assigned by the ECtHR. Among the decisions taken by governments, only those concerning changes in laws or the application of laws were noted. The decisions concerning the “dissemination of the Court's judgement” were not noted.

## Results

### Case-Law of the ECtHR

Among the 22,536 judgements and 26,887 decisions of the Court between December 31, 1960, and December 31, 2019, the research in the HUDOC database identified 174 cases of case law concerning psychiatric commitment. This case-law included 56 decisions on the admissibility of applications, and 118 judgements of the Court concerning violations of articles of the Convention.

Table 1 gives the number of ECtHR decisions and judgements classified by country and by degree of importance. The 56 decisions, taken by a Chamber or by the Commission (up to 31 October 1999), consisted of 25 applications declared inadmissible, 15 applications declared partially admissible and partially inadmissible, and 16 applications declared admissible. The 118 judgements were divided into 12 key cases, 14 level 1 cases, 63 level 2 cases, and 84 level 3 cases.

**Table 1:** ECtHR judgements and decisions by country and by level of importance.

**Country**	**Decisions**	**Judgements**	**Total**	**Key cases**	**Level 1 cases**	**Level 2 cases**	**Level 3 cases**
Albania		1	1			1	
Andorra							
Armenia							
Austria	5	2	7		1	1	6
Azerbaijian							
Belgium	2	20	22	1	3	5	13
Bosnia Herzegovina		3	3			3	
Bulgaria	3	8	11	2		1	8
Croatia		2	2			1	1
Cyprus							
Czech Republic		3	3			3	
Denmark							
Estonia							
Finland	2	1	3	1			2
France		2	2			2	
Georgia							
Germany	6	10	16	2	3	5	6
Greece		2	2				2
Hungary		2	2			1	1
Iceland	1	1	2		2		
Ireland	1		1				1
Italy	1	1	2		1		1
Latvia		5	5			4	1
Liechtenstein	1		1	1			
Lithuania		2	2			1	1
Republic of Moldova		3	3			2	1
Monaco							
Montenegro							
Netherlands	9	10	19		3	5	11
North Macedonia		1	1			1	
Norway		1	1			1	
Poland	5	5	10	1		2	7
Portugal							
Romania		6	6		1	4	1
Russia	2	13	15	1		10	4
San Marino							
Serbia							
Slovakia		2	2			2	
Slovenia							
Spain							
Sweden	2		2				2
Switzerland		4	4			2	2
Turkey		1	1			1	
Ukraine		5	5			3	2
United Kingdom	16	6	22	3	2	3	14

The key cases are summarized in Table 2 and the level 1 cases are summarized in Table 3. It can be seen that the requests relating to the deprivation of liberty during psychiatric commitment can also relate to other aspects of Human Rights, in particular to Article 3 of the Convention (prohibition of inhuman and degrading treatment) and to Article 6 of the Convention (right to a fair trial). In the event of a violation of an article of the Convention, two situations may arise: either there is a national law that protects the rights of the patient, but this law has not been respected; or the national law is not in conformity with the principles of the Convention for the protection of the rights of the patient. It is especially in these fault situations that the judgement of the Court leads to the obligation of the government of the country concerned to amend its national legislation. The non-conformity of the national law with the principles of the Convention can relate to very different aspects, but it often acts of a failure in the establishment of the statement of mental illness or a defect in the possibilities for the patient to appeal against his deprivation of liberty or even an excessively long duration of the procedures.

**Table 2:** ECtHR pilot case law concerning psychiatric commitment.

**References**	**ECtHR decisions**	**Concerned articles and ECtHR interpretations**
Frommelt v. Liechtenstein	Inadmissible	5-1-c: The transfer to a psychiatric establishment in another state was in accordance with national law.
49158/99		3 : The applicant was not subjected to inhuman and degrading treatment
Hutchison Reid v. The United Kingdom	No violation	5-1-e: Maintaining the psychiatric commitment if the disease is incurable is not a violation of human rights.
50272/99	Non conformity of NL	5-4: It is not up to the patient to prove that he does not have a mental disorder.
	Violation of the NL	5-4: The excessive length of time for examining the appeal was not admissible.
H.L. v. The United Kingdom	Non conformity of NL	5-1: There is a lack of any fixed procedural rules by which the admission and detention of compliant incapacitated persons is conducted.
45508/99		5-4: There is a lack of procedures allowing an incapacitated patient to appeal the deprivation of his liberty.
Ilnseher v. Germany	No violation	5-1-e: The applicant's mental disorder was such that it warranted his preventive detention as a person of unsound mind.
10211/12 and 27505/14		7: The preventive detention ordered because of and in order to address the mental condition could not be considered a “penalty”.
Kolanis v. The United Kingdom	No violation	5-1: The continued compulsory detention pending adequate discharge conditions does not constitute a violation of human rights.
517/02	Violation of NL	5-4: The length of the proceedings was excessive and failed to meet the “reasonable time”.
	Non conformity of NL	5-5: There was a lack of enforceable rights to compensation.
Rooman v. Belgium	Violation of NL	3: The continued detention without appropriate medical support for 13 years and without any realistic prospect of change constituted degrading treatment.
18052/11	No violation	3: The organization of even incomplete care makes it possible to rule out the notion of inhuman and degrading treatment.
	Violation of NL	5-1: There is an obligation of means in overcoming a linguistic obstacle to the treatment of the mental disorders suffered by an individual in compulsory confinement.
	No violation	5-1: The efforts of the authorities to provide adequate care make it possible to rule out a violation of Art. 5-1 during a certain period of the deprivation of liberty.
Shtukatourov v. Russia	Violation of NL	6-1: The proceedings of legal capacity deprivation were not fair.
44009/05		5-1-e: It has not been reliably shown that the applicant suffered from mental illness at the time of his psychiatric commitment.
		34: The applicant was prohibited from meeting with his lawyer during his psychiatric commitment.
	Non conformity of NL	8: National law does not allow a partial limitation of legal capacity adapted to each individual case.
		5-4: National law does not provide for any judicial review of psychiatric commitment.
Stanev v. Bulgaria	Non conformity of NL	5-1: There is a lack of lawfulness of placement in a social care home for persons with mental disorders.
36760/06		5-4: There is a lack of remedies to challenge the lawfulness of placement in a social care home for persons with mental disorders.
		5-5. There is a lack of means for the applicant to avail himself of a right to compensation for the unlawful deprivation of his liberty.
		6-1. There is a lack of direct access to a court for a person seeking the restoration of his legal capacity.
	Violation of NL	3: The living conditions in a social care home for persons with mental disorders were comparable to inhuman and degrading treatment.
Stork v. Germany 38033/02	Violation of NL	6-1: The length of the proceedings was excessive and failed to meet the “reasonable time.”
Varbanov v. Bulgaria	Non conformity of NL	5-1-e: The domestic law does not provide the required protection against arbitrariness since it does not require a medical opinion.
31365/96	Non conformity of NL	5-4: The applicant was deprived of his right to have the lawfulness of his detention reviewed by a court.
Witold Litwa v. Poland 26629/95	Violation of NL	5-1-e: There is no less rigorous measure than the detention of a drunk and almost blind person.
X v. Finland	Non conformity of NL	5-1: The national law did not provide adequate safeguards against arbitrariness.
34806/04		8: There is a lack of judicial remedies against forced medication.

*NL, National Law*.

**Table 3:** ECtHR level 1 case law concerning psychiatric commitment.

**References**	**ECtHR decisions**	**Concerned articles and ECtHR interpretations**
Aerts v. Belgium 61/1997/845/1051	Non conformity of NL	5-1: There was an excessive length of provisional detention in an establishment that was not appropriate for mentally ill persons.
	No violation	5-4: The applicant had access to a court to appeal against his psychiatric commitment.
	Violation of NL	6-1: The applicant has not been allowed to use legal aid to appeal on points of law.
	No violation	3: The living conditions on the psychiatric wing have not been classified as inhuman or degrading.
Ashingdane v. The United Kingdom 8225/78	No violation	5-1: The applicant's poor mental health has been properly established and he was justifiably deprived of liberty.
	No violation	5-4: A request for a change in hospital category is not subject to appeal to a court.
	No violation	6-1: The applicant's right of access to a court was not denied.
Bergmann v. Germany 23279/14	No violation	5-1-e: The preventive detention of a mental health patient in a center offering appropriate medical care was in compliance with domestic law and was not arbitrary.
		7-1: The retrospective extension of preventive detention intended to secure medical and therapeutic treatment was not a violation.
Brand v. The Netherlands 49902/99	Non conformity of NL	5-1: A delay of 6 months in the admission of a person to a custodial clinic was not acceptable.
Filip v. Romania 41124/02	No violation	3: The facts were not sufficiently well-established to support a violation.
	Violation of NL	3: There was a lack of a thorough and effective investigation into the applicant's allegation of ill treatment in the psychiatric hospital.
	Violations of a national law	5-1: The applicant's mental illness has not been conclusively established and the consideration of his complaint was delayed.
		5-4: There had been no review of the lawfulness of the applicant's detention
Hafsteinsdottir v. Iceland 40905/98	Non conformity of NL	5-1: The exercise of discretion by the police and the duration of the deprivation of liberty had been governed by administrative practice alone and not by a legal framework.
Haidn v. Germany 6587/04	Violation of NL	5-1: There was an indefinite preventive detention of a mentally ill person following the completion of prison term.
	No violation	3: The minimum level of severity required for inhuman or degrading treatment or punishment had not been attained during the detention.
Herczegfalvy v. Austria 10533/83	No violation	5-1: The judicial authorities did not fail to observe the procedures of national law when ordering the placement of the applicant and when confirming the measures.
	No violation	5-3: The length of the two periods of pretrial detention had not exceeded a “reasonable time.”
	Violation of NL	5-4: Two of the three decisions taken in the context of the automatic periodic review of the lawfulness of the deprivation of liberty did not occur at reasonable intervals.
	No Violation	3: Forced feeding, forced neuroleptic treatment, isolation and securing to a safety bed were justified by the requirements of the medical treatment.
	No violation	8: The medical treatment and force-feeding were justified because the patient was entirely incapable of making decisions for himself.
	Non conformity of NL	8: Sending all the applicant's letters to his curator for review constituted interference with the exercise of the applicant's right to respect for their correspondence.
	Violation of NL	10: The restriction on access to information during hospitalization was arbitrary.
Luberti v. Italy 9019/80	No violation	5-1: The applicant's mental health and dangerousness were properly established, and he was rightly deprived of his liberty.
	Violation of NL	5-4: The judicial authorities did not rule speedily on the requests to lift the psychiatric confinement.
L.B. v. Belgium 22831/08	Violation of NL	5-1-e: A person of unsound mind was detained in psychiatric prison wings for seven years despite the authorities' advice to place him in an institution suited to his mental state.
Morsink v. The Netherlands 48865/99	Non conformity of NL	5-1: Preplacement detention in a remand center for more than 15 months pending transfer to a custodial clinic was not acceptable.
Winterwerp v. The Netherlands 6301/73	No violation	5-1: The psychiatric confinement constituted the lawful detention of a person of unsound mind.
	Non conformity of NL	5-4: The various bodies that ordered or authorized the psychiatric detention did not possess the characteristics of a court.
	Non conformity of NL	6-1: The mental illness from which a person suffers cannot justify the complete lack of the right of access to a court.
W.D. v. Belgium 73548/13	Violation of NL	3: A person was deprived of their liberty for more than 9 years without any appropriate care or any realistic prospect of rehabilitation.
		5-1: There was unlawful deprivation of liberty and continued detention.
		5-4, 13: There was no effective remedy in order to complain about the conditions of detention.
X v. The United Kingdom 7215/75	No violation	5-1: The emergency psychiatric confinement met the minimum conditions to be lawful.
	Non conformity of NL	5-4: National procedures did not allow the lawfulness of the psychiatric commitment to be challenged in court.

*NL, National Law*.

### Effects of ECtHR Judgements and Decisions on Domestic Laws

The effects of ECtHR judgements in key cases and level 1 cases are summarized in Tables 4, 5, respective. It is possible to note that Austria, Belgium, Bulgaria, Germany, Iceland, the Netherlands, Romania, and the United Kingdom have modified their national laws concerning psychiatric commitment as direct consequences of ECtHR judgements. The modifications varied according to the domestic laws, but it is possible to note that the changes were always in the direction of improvement with respect to the rights of the patients, in particular regarding the aspects of the initial conditions of the commitment, of the right of appeal against the placement decision, of the reduction in the length of the procedures, and of the improvement of the living conditions and treatments of interned persons.

**Table 4:** Consequences of the pilot case law of the ECtHR on national legislations (text extracted from the Council of Europe HUDOC EXEC database).

**References**	**Measures taken by the concerned State**
Hutchison Reid v. the United Kingdom 50272/99	The Mental Health (Public Safety and Appeals) Scotland Act of 1999 makes it clear that in proceedings regarding the review of the detention of a mentally handicapped person, the burden of proof lies on the authorities. The application of the above legislation by the Sheriff Court is demonstrated. Furthermore, the number of judges in the Court of Session has been increased to speed up proceedings.
H.L. v. The United Kingdom 45508/99	In England and Wales, the Deprivation of Liberty Safeguards (DOLS) in Section 50 and Schedules 7, 8, and 9 introduced a series of procedural safeguards to the Mental Health Act of 2007. A code of practice was published on 28/08/2008. No amendments were required to the Scottish legislation. In Northern Ireland, in October 2010, the Department of Health, Social Services and Public Safety published further guidance. The Health and Social Board, which commissions all health and social care services in Northern Ireland, monitors the application of the guidance through biannual reports and an assurance process of judicial review is available to challenge any failure to apply the guidance by public authorities. A Mental Capacity Bill will be introduced into the Northern Ireland Assembly in 2015 and enacted before March 2016.
Kolanis v. The United Kingdom 517/02	In 2002, there was a development in the domestic case-law with respect to section 73 of the Mental Health Act of 1983.
	An enforceable right to compensation for violation of Article 5, paragraph 4 was introduced by the Human Rights Act of 1998, which entered into force in 2000.
Rooman v. Belgium 18052/11	Provision of places for internees in non-prison institutions (new facilities or those already part of the mainstream care provision network); measures to promote the “care pathway”; Law of 5 May 2014 on internment; the framing of support policies in the federated entities; the original functions of enhanced consultation and dialogue between all the relevant authorities, the social defense sections and psychiatric annexes have been maintained.
Shtukaturov v. Russia 44009/05	The measures taken by the defendant state remain unknown.
Stanev v. Bulgaria	An amended version of the 1998 Social Assistance Act (hereafter “the 1998 Act”) entered into force early in 2016. It excludes involuntary placement and introduces different procedures for voluntary placement in an institution or in a “residential service” in the community depending on whether the person is under partial or full guardianship.
36760/06	A draft law on physical persons and support measures was introduced before the Bulgarian Parliament on 4 August 2016. It foresees the abolition of full and partial guardianships and their replacement by support measures.
	On 27/10/2017, the Code of Civil Procedure was amended to give persons under partial guardianship direct access to a court to request the restoration of their legal capacity.
Stork v. Germany 38033/02	The Act on Legal Redress for Excessive Length of Court Proceedings and of Criminal Investigation Proceedings entered into force 1 year after the pilot judgement became final on 3 December 2011.
Varbanov v. Bulgaria 31365/96	According to the new Health Act of 2005, only a court is competent to order an expert opinion and, if necessary, to order confinement with the goal to obtain a psychiatric examination following a public hearing at which the person concerned, assisted by counsel and a psychiatrist, must be heard. The decision may be appealed. The judgement was published, translated and disseminated.
Witold Litwa v. Poland 26629/95	Copies of the judgement translated into Polish had accordingly been sent out to the police and staff of all sobering-up centers under the supervision of local self-governments, together with a circular letter from the Ministry of Internal Affairs.
X v. Finland 34806/04	The authorities expressed their intention to adopt amendments to the Mental Health Act. Pending the adoption of the legislative reform, the Ministry of Social Affairs and Health gave instructions concerning the involuntary treatment, including the medication of a patient.

**Table 5:** Consequences of the level 1 case law of the ECtHR on national legislations (text extracted from the of the Council of Europe HUDOC EXEC database).

**References**	**Measures taken by the concerned State**
Aerts v. Belgium	The legal aid office amended its practice in September 1998 and the system of legal aid at the *Cour de Cassation* was subsequently amended by Parliament in November 1998 (Law No. 98/3417) with the goal of placing destitute people or those with insufficient means on equal footing with people with sufficient means.
61/1997/845/1051 49902/99	*Ad hoc* measures to increase the number of places available in social protection centers were already being introduced. In July 1999, the waiting time for transfers was 2 months.
Brand v. The Netherlands	In its judgement on 21/12/2007, the Dutch Supreme Court held that preplacement detention exceeding 4 months was unlawful. From 2006 to 2011, the custodial clinics' capacities were enlarged. In 2013, the average waiting time amounted to 100 days.
Filip v. Romania 41124/02	The new Code of Criminal Procedure of 2014 brought significant changes to non-voluntary confinement for compulsory treatment and committal to a psychiatric institution for expert examination during criminal proceedings.
Hafsteinsdottir v. Iceland 40905/98	In the new Code of Criminal Procedure of 1992, the provisions on arrest in the interests of public peace and order were removed from the Code of Criminal Procedure and included in the new Police Act of 1997.
Haidn v. Germany 6587/04	The Act to Effect Implementation under Federal Law of the Distance Requirement in the Law Governing Preventive Detention entered into force on 01/06/2013. This amended the relevant provisions of the criminal code and established guiding principles regarding the treatment and placement of preventive detainees. The Länder, responsible for the execution of preventive detention within Germany's federal structure, modified their laws accordingly.
Herczegfalvy v. Austria	Article 51, paragraph 1 of the Hospitals Act was deleted and replaced with more precise provisions.
10533/83	As of 1 January 1991, Article 34 of the Act on the Placement of the Mentally Ill provides that the correspondence between a patient and his counsel may no longer be hindered and that the patient's right to correspond with other persons may only be limited to the extent necessary to protect the patient's health. As of 1 January 1994, these provisions also apply to convicted persons placed in mental hospitals in accordance with a new Article 167.a in the Law on the Enforcement of Sentences.
	The new Article 167.a also provides that persons serving their sentences in mental hospitals may see their contacts with the outside world restricted only to the extent that such contacts would create a risk that the person concerned would commit new crimes.
	As of 1 January 1994, Article 58 of the Law on the Enforcement of Sentences provides that the detainees should have access to, inter alia, reading material and to television and radio.
Luberti v. Italy 9019/80	The measures taken by the defendant state are unknown.
L.B. v. Belgium (and other cases)	The new law of 5 May 2014 on internment entered into force on 1 October 2016. The law included the following:
22831/08	The provision of places for internees in non-prison institutions;
	Measures to promote the “care pathway”
	The framing of support policies in the federated entities; Enhanced consultation and dialogue between all the relevant authorities
Morsink v. The Netherlands 48865/99	In its judgement on 21/12/2007, the Dutch Supreme Court held that preplacement detention exceeding 4 months was unlawful. From 2006 to 2011, the custodial clinics' capacities were enlarged. In 2013, the average waiting time was 100 days.
Winterwerp v. The Netherlands 6301/73	On 5 February 1980, in the Second Chamber of Parliament, the Government introduced a revised bill on “special admissions to psychiatric hospitals” that stated that in all cases of involuntary admission to psychiatric hospitals, prolongation of admission or requests for dismissal, the patient has the right to be heard by a court. In the new bill, Article 32 of the Mentally Ill Persons Act (automatic loss of the patient's administration of his property as a result of his involuntary admission to a psychiatric hospital) has been deleted.
W.D. v. Belgium (and others cases)	The new law of 5 May 2014 on internment entered into force on 1 October 2016. The law included the following:
73548/13	The provision of places for internees in non-prison institutions; Measures to promote the “care pathway”; The framing of support policies in the federated entities; Enhanced consultation and dialogue between all the relevant authorities.
X v. The United Kingdom 7215/75	Amendments designed to remedy the deficiency in domestic law found by the European Court were inserted into the Mental Health (Amendment) Bill. These amendments have been enacted and entered into force on 30 September 1983.

It should also be noted that not all countries follow the decisions of the judgments of the Court. Russia is notorius for changing nothing, despite the convictions by the Court. It is difficult to know precisely, by reading the requests and judgments, the reality of what is happening in these countries for patients interned in psychiatric hospitals. The action of organizations which intervene directly in places of detention, such as the CPT, is therefore essential in denouncing the persistence of human rights violations.

## Discussion

This study of the case-law of the ECtHR shows that 31 of the 45 countries that have ratified the Convention have been affected by a decision or a judgement of the Court concerning psychiatric commitment. The number and nature of changes required by the Court's judgements, however, vary widely from country to country. In fact, there are multiple reasons why a country is the subject of decisions or judgements of the ECtHR, and there is no direct relation to the quality of their legislation concerning psychiatric commitment. For example, the size of the country's population and the time since it has ratified the Convention are obvious factors. It is also possible to observe that countries with very developed legal systems and where the defense of the rights of the citizens is well represented, such as Belgium, the Netherlands, Germany, or the United Kingdom, are subject to many applications to the Court and therefore more decisions or judgements (11). It is therefore irrelevant to compare countries with one another in terms of the number of judgements of the ECtHR. The activity of the ECtHR must be understood as a global process of improving jurisdiction throughout the geographical area covered by the activity of the Court. The study of the ECtHR case law and of the database of the Department for the Execution of judgements of the ECtHR of the Council of Europe clearly shows the direct and concrete favorable effects of the ECtHR on the rights of patients affected by psychiatric confinement (12). Reading the proceedings of the Court also reveals the essential role of dedicated and committed counsel in ensuring the representation of applicants and asserting their rights (13).

However, it would be wrong to believe that the influence of the Court is limited to these direct effects. From a historical point of view, during the 60 years from the first judgements of the Court in 1960 until 2019, many changes have been made in the laws of Council of Europe countries concerning psychiatric commitment without these countries being directly affected by judgements of the Court but drawing inspiration from the case law of the ECtHR (14). First, the key cases and the level 1 cases serve as references for legislators when they design a change in law or create new laws. Second, on the basis of its own case law, the ECtHR gives guidelines on the main points that should structure national laws. Regarding psychiatric commitment, the ECtHR's guidelines were updated in December 2019 in the Guide to Article 5 of the European Convention on Human Rights (15):

- The detention of persons of unsound mind is an exception to the general principle of Art. 5 of the Convention. An essential point of the case law is therefore to determine what the term person of “unsound mind” means. The Court has always been careful not to depend on local or specific definitions of a law or a social context (Rakevich v. Russia, Petschulies v. Germany, Ilnseher v. Germany).
- The ECtHR defines three minimum conditions for an individual to be deprived of his liberty as being of “unsound mind”:
  ➢ The first condition is that unless it is an emergency situation, an objective medical expert must establish that the individual is of unsound mind (Ruiz Rivera v. Switzerland, S.R. v. the Netherlands). If a medical examination of the person is not possible, the Court accepts that the expert can make a determination based on the case file (Constancia v. the Netherlands). The mental disorder must be of a certain seriousness to be considered a “true” mental disorder, that is, it must necessitate treatment in an institution appropriate for mental health patients (Glien v. Germany, Ilnseher v. Germany, and Petschulies v. Germany). The existence of the mental disorder must be conclusively established as soon as the person concerned is in psychiatric commitment (Ilnseher v. Germany, O.H. v. Germany). The evolution of mental health after placement must be taken into account and the medical reports on which the authorities are based must be sufficiently recent (Ilnseher v. Germany, Kadusic v. Switzerland).
  ➢ The second condition is that the individual's mental disorder must justify the compulsory confinement. In the case-law of the Court, this condition concerns not only the need for treatment but also the need for control and supervision to prevent the person from harming himself or other persons (Ilnseher v. Germany, Hutchison Reid v. the United Kingdom, and N. v Romania).
  ➢ The third condition is that the trouble must persist throughout the period of the deprivation of the individual's liberty. This means that as soon as the mental disorder is no longer present, the person concerned must be released. However, the Court recognizes that the authorities may have a short period of time to examine whether the detention can be lifted (Luberti v Italy). Psychiatric commitment, however, cannot be maintained for administrative reasons (R.L. and M.-J.D. v. France).
- The place of psychiatric commitment gave rise to the case-law of the Court. Normally, the placement of a person of unsound mind should be carried out in “a hospital, a clinic or an appropriate institution authorized for the detention of such persons” (L.B. v. Belgium, Ashingdane v. the United Kingdom, and O.H. v. Germany). However, if the time limit is short, the psychiatric commitment will take place temporarily in an establishment not specifically designed for this kind of placement (Pankiewicz v. Poland, Morsink v. the Netherlands, and Brand v. the Netherlands).
- The Court has regularly noted that the administration of suitable therapy is one of the legal conditions justifying the deprivation of liberty of a person of unsound mind. The psychiatric commitment has the function of treating or curing the patients on the one hand and, if necessary, reducing or managing their dangerousness on the other hand (Rooman v. Belgium). These two functions constitute the concept of “appropriate institution.”
- The application of Article 5-1-e of the Convention includes procedural safeguards related to the procedure of involuntary hospitalization (M.S. v. Croatia, n°2). A fair procedure must allow the interned person to have access to a means of protection against arbitrariness (V.K. v. Russia, X. v. Finland). It therefore seems particularly important that a person in psychiatric commitment should have access to a court and be heard either in person or through a representative. Each interned person must have access to legal assistance for any procedure relating to the deprivation of liberty (N. v. Romania). The Court noted that legal assistance should not only be formal, but it should be effective and controlled by competent domestic courts [M.S. v. Croatia (n°2), V.K. v. Russia].
- Concerning the application of Article 5-1-e to situations of the deprivation of liberty of people under the influence of alcohol, the Court considered that the term “alcoholics” concerns not only people dependent on alcohol but also people whose consumption of alcohol poses a danger to themselves and to others. (Kharin v. Russia, Hilda Hafsteinsdottir v. Iceland). However, according to the Court, Article 5-1-e does not allow the deprivation of liberty of a person solely because he or she consumes alcohol (Petschulies v. Germany, Witold Litwa v. Poland).

The case law of the ECtHR determines the profile of the judicial procedures allowing the internment of people of unsound mind when it is necessary while preserving the rights of those concerned. However, it is not the role of the Court to determine an ideal psychiatric commitment procedure that could be applied universally. The case law of the ECtHR is constantly in line with the historical and cultural particularities of the States. However, inspired by the work of the ECtHR and that of the CPT, the Committee of Ministers of the Council of Europe in 2004 issued recommendations to member States concerning the respect of people with mental disorders, and in Articles 17–25 they included recommendations concerning the involuntary hospitalization of people suffering from mental disorders (16). These articles set out the conditions under which a person may be placed in a hospital involuntarily, the essential remedies and the conditions for the termination of the placement. Several European countries, such as Austria, France, Germany, Italy and Spain, have modified or amended their laws on psychiatric commitment according to the directives of the Council of Europe (14). It is therefore appropriate to consider that the case-law of the ECtHR has, in addition to the direct effects due to judgements, indirect effects by inspiring changes in the legislation of many countries. Finally, in a movement of reciprocal influence, it can be noted that the ECtHR itself has cited in recent judgments (Asalya v. Turkey, Kuttner v. Australia) Article 14 of the Convention on the Rights of Persons with Disabilities (17).

## Conclusion

Whatever the country and whatever the time, psychiatric involuntary hospitalization is and will remain a situation of conflict and confrontation between an individual and a State. Psychiatric commitment is a paradigmatic expression of the question of human rights expressed in the field of psychiatry. A just law must strike a balance between the rights of the individual and the protection of society. The correctness of the law is essential for respecting patients' rights and therefore for safeguarding ethics in the practice of caregivers and hospital doctors. The case law of the ECtHR is a constant source of inspiration for improving the laws on psychiatric commitment and for the proper implementation of this law in the practice of care and treatment. Other sensitive areas of psychiatry, such as forced treatment or measures restricting freedom, are also the subject of an ECtHR case law that is often not well known.

## Data Availability Statement

The original contributions presented in the study are included in the article/supplementary material, further inquiries can be directed to the corresponding author/s.

## Ethics Statement

Written informed consent was not obtained from the individual(s) for the publication of any potentially identifiable images or data included in this article.

## Author Contributions

All authors have made a substantial, direct and intellectual contribution to the work, and approved it for publication.

## Conflict of Interest

The authors declare that the research was conducted in the absence of any commercial or financial relationships that could be construed as a potential conflict of interest.
